# Chromosomal quality control in hPSCs: A practical guide to SNP array analysis with GenomeStudio

**DOI:** 10.3389/fcell.2025.1599923

**Published:** 2025-07-01

**Authors:** Josephine Haake, Laura Steenpass

**Affiliations:** ^1^ Department of Human and Animal Cell Lines, Leibniz Institute DSMZ – German Collection of Microorganisms and Cell Cultures GmbH, Braunschweig, Germany; ^2^ Zoological Institute, Technical University Braunschweig, Braunschweig, Germany

**Keywords:** SNP array analysis, GenomeStudio, chromosomal stability in hPSCs, quality control of hPSCs, B-allele frequency, log R ratio

## Abstract

Human pluripotent stem cells (hPSCs) are important tools in preclinical research and disease modeling. Valid results can only be obtained using thoroughly quality-controlled hPSCs, which includes ensuring chromosomal stability. Chromosomal aberrations, which frequently arise during reprogramming, gene editing, or maintenance cultivation, can compromise the utility of these cells in research and therapeutic applications. Although traditional G-banding remains a valuable genome-wide analysis method, its limited resolution necessitates complementary approaches. SNP array analysis offers a high-resolution alternative, providing a more detailed genomic overview. We present a practical and user-friendly guide for detecting chromosomal aberrations using Illumina’s GenomeStudio, offering an easy-to-follow protocol to simplify quality control workflows for researchers with minimal bioinformatics expertise. Although SNP array analysis for hPSC quality control is not novel, this step-by-step guide highlights critical quality control metrics, thresholds, and values, streamlining the process to make it more accessible and efficient for broader adoption. In 32 hPSCs, we identified chromosomal aberrations in nine, including the frequently reported gain of 20q11.21—a common anomaly in hPSC cultures. Examples from our routine practices underscore the importance of monitoring chromosomal integrity. This guide serves as a practical resource for standardizing and enhancing quality control processes, ensuring the genomic stability of hPSCs for research and clinical applications.

## Introduction

Reprogramming, *in vitro* cultivation, and gene editing increase the genetic instability of hPSCs, potentially resulting in chromosomal abnormalities. It has been reported that a genetically abnormal clone can completely overtake a culture in less than five passages (Bai et al., 2013; Baker et al., 2007; Tigges et al., 2021). Studies over the last two decades revealed a bias in the genetic changes acquired in hPSCs, with common karyotypic abnormalities involving trisomy of chromosome 12, 17, or X or the amplification of chromosome 1, 12p, 17q, or 20q11.21 (Amps et al., 2011; Baker et al., 2016; Draper et al., 2004). These variants could significantly impact the therapeutic and research use of hPSCs, affecting the efficiency of differentiation protocols, functionality of differentiated cells, or the safety of cell replacement therapies (Andrews et al., 2017; Halliwell et al., 2020; Price et al., 2021). Therefore, monitoring chromosomal stability and alterations is mandatory for good cell culture practice and the generation of hPSC lines and should be included in the quality control panel.

Karyotyping by G-banding is the gold-standard method for monitoring hPSC cultures for genomic changes, offering a whole-genome overview in a single assay. Structural chromosomal aberrations, like translocations, can only be detected by G-banding. However, G-banding has several practical limitations, including the requirement for living cells and the need for high-level expertise. Its relatively low resolution, detecting only larger-scale aberrations of 5–10 Mb or greater limits its ability to identify smaller genetic alterations below this threshold (Avery et al., 2013; Steventon-Jones et al., 2022). Single-nucleotide polymorphism (SNP) array platforms offer a potential alternative with improved detection of copy number variants and thereby chromosomal abnormalities. SNPs are single-base variations in the DNA sequence and are one of the most common types of genetic variation, which not necessarily cause genetic diseases. The two possible alleles of a SNP are typically labeled as A and B. As individuals inherit one allele from each parent, their genotype at an SNP site is AA, AB, or BB (LaFramboise, 2009). Over 600 million SNPs have been identified across the human genome in the global population (Sherry et al., 2001). SNP arrays are DNA microarrays designed with probes targeting specific biallelic SNP positions. These arrays are hybridized with fragmented DNA to determine the specific alleles present in the sample (Kruglyak, 1997; LaFramboise, 2009; You et al., 2018).

Illumina’s BeadArray technology uses silica microbeads coated with multiple copies of 50-mer oligonucleotide probes that target specific SNP loci in the genome, using a two-color system for detection. G/C nucleotides are labeled with biotin, whereas A/T nucleotides are labeled with 2,4-dinitrophenol (DNP). Detection is based on fluorophore-labeled streptavidin for C/G and fluorophore-labeled antibodies for A/T. The tagged nucleotides are detected through an immunohistochemical sandwich assay, resulting in red fluorescence for A and T and green fluorescence for C and G. To detect each possible SNP combination, two probe designs are used in the BeadArray: the Infinium type I design, which detects the relatively less common A/T and G/C SNPs (17% of all SNPs), and the Infinium type II design, which is used for the more common SNPs (83% of all SNPs), including A/G, A/C, T/C, and T/G. The type I design is based on two allele-specific primer extension (ASPE) probes that include the SNP position. The DNA fragment hybridizes to the probe in an allele-specific manner and is then extended by the nucleotide that follows the SNP. The position of the specific probe on the array is used to decode the genotype of the SNP: a green signal at only one position indicates homozygosity for G or C, whereas a green signal at both positions indicates heterozygosity for G/C. Conversely, a red signal at one position indicates homozygosity for A or T, and a red signal at both positions indicates heterozygosity for A/T SNPs. The type II design, on the other hand, is based on a single-base extension (SBE) probe that terminates one nucleotide before the SNP position. DNA fragments hybridize to the probes, which are then extended by a single nucleotide complementary to the base at the SNP site. A red signal indicates homozygosity for A or T, a green signal indicates homozygosity for G or C, and a yellow signal, resulting from the combination of red and green, indicates heterozygosity for A/C, A/G, T/C, or T/G (Guo and Jamison, 2005; Steemers et al., 2006; Steemers and Gunderson, 2007). The fluorescent signal corresponding to the incorporated nucleotide allows detection of chromosomal abnormalities such as deletions, gains, and loss of heterozygosity (LOH) larger than 350 kb with high sensitivity using GenomeStudio for analysis (Shen et al., 2005).

SNP array can be used for molecular karyotyping to detect chromosomal aberrations in hPSC culture (Canham et al., 2015; Rauch et al., 2004). Copy number variations (CNVs), which show increases or decreases in chromosomal copies for a given region in the genome, can be sensitively detected in a genome-wide manner. In addition to detecting copy number losses (CNL-LOH) and duplications, a unique feature of SNP array-based copy number analysis is its ability to sensitively identify copy-neutral loss of heterozygosity (CN-LOH), which cannot be identified by metaphase karyotyping. CN-LOH represents an abnormal allelic status in which both alleles originate from a single parent, due to either uniparental disomy or chromosomal loss and duplication of the remaining allele (Sato-Otsubo et al., 2012). However, SNP arrays cannot detect balanced translocations, and their ability to identify sub-clonal populations or sidelines within a cell line is limited and depends on factors such as the proportion of the abnormal cell population and the resolution of the array (Steventon-Jones et al., 2022).

Illumina’s GenomeStudio with the cnvPartition plug-in provides a fast and easy tool for analyzing SNP array data to identify genetic instabilities during stem cell culture quality control. In addition to specific quality assessments for hPSCs, such as differentiation potential and the expression of pluripotency markers, detecting chromosomal aberrations is a critical component of the quality control panel. Whereas G-banding analysis identifies larger structural or numerical chromosomal abnormalities, smaller aberrations may remain undetected using this method. Therefore, SNP array analysis contributes an additional tool for proof of genomic stability. We provide a practical guide for detecting these aberrations in hPSCs using GenomeStudio to underscore the importance of chromosomal integrity checks during hPSC research, particularly in situations where bioinformatics support is limited.

## Materials and methods

### Cell lines and cultivation

All experiments were performed in accordance with relevant guidelines and regulations. The use of hESCs has been reviewed and approved by the Central Ethical Review Board for Stem Cell Research at the Robert Koch Institute, Berlin, Germany (Az.3.04.02/0167). Human PSCs were maintained at 37°C with 5% CO_2_ and 21% O_2_ in specific media and matrix, as detailed in Supplementary Table S1. Cells were passaged at a 1:6 ratio using the appropriate passage method every 5 to 6 days. A list of hPSCs is provided in Supplementary Table S1.

**TABLE 1:** Chromosomal aberrations detected via SNP array in hPSC lines during in-house quality control.

Cell-line	Aberration	Location	Timing of detection	Reported in hPSCs/known association
ZIPi015-K	Gain	20q11.21	During cell culture	Common aberration in hPSCs (Amps et al., 2011)
GM24581	Gain	20q11.21	During cell culture	Common aberration in hPSCs
STBCi101-A	Gain	20q11.21	During cell culture	Common aberration in hPSCs
DSMZi002-C-11	Gain	20q11.21	After reprogramming	Common aberration in hPSCs
DSMZi017-A	CN-LOH	9q (whole long arm)	During cell culture	
	Gain	10q11.22	Suspected parental origin	Common CNV (DGV comparison)
	Deletion	15q11.2-q12	Parental origin	Angelman syndrome related
H9	Gain	7q11.21	Adaptation to cell culture	Frequently observed in hPSCs (Baker et al., 2016; Baker et al., 2007)
	Gain	14q23.3	Adaptation to cell culture	Frequently observed in hPSCs (Baker et al., 2016; Baker et al., 2007)
	Deletion	16p11.2		
RBi001-A	CN-LOH	4q33-q34.3		
WTSIi021-A	Deletion	7q31.1, 7q31.33	Adaptation to cell culture	Frequently observed in hPSCs (Andrews et al., 2022)
ZIPi015-K-2-C-3	CN-LOH	15q	After CRISPR/Cas9 gene editing	Gene editing on chr15

### Karyotype analysis

Cells were treated with 0.04 μg/mL colcemid (Gibco by Thermo Fisher Scientific, Darmstadt, Germany) for 2 h. Single-cell suspensions were produced using Accutase (Sigma-Aldrich by Merck, Darmstadt, Germany) and incubated in a hypotonic solution (1:1 0.075M KCl:0.9% sodium citrate) for 60 min at 37°C. Cells were fixed with a 1:3 acetic acid:methanol solution. G-banding analysis was performed using Stem Genomics (Montpellier, France). At least 20 metaphases were structurally evaluated with a chromosome band resolution between 300 and 500 band levels.

### SNP array

Genomic DNA was extracted using the QIAamp DNA Blood Mini Kit (Qiagen, Hilden, Germany) and processed on the Global Screening Array v3.0 (GSAMD24v3-0, Illumina, Inc. San Diego) by LIFE & BRAIN GmbH (Bonn, Germany). SNP calling was performed using GenomeStudio V2.0.5 with a GenCall threshold of 0.2. CNV analysis was performed using cnvPartition 3.2.0.

### Key values for CNV detection using GenomeStudio

There are different values and thresholds which are necessary to be mentioned using GenomeStudio to ensure quality of SNP array data and reliability of CNV detection.

The call rate represents the percentage of SNPs that are successfully assigned to a genotype or copy number state out of the total number of probes on the array. In the literature, a call rate between 95% and 98% is generally considered acceptable (Guo et al., 2014). For quality control of hPSCs in research, we recommend adopting the lower threshold of 95% as the goal is to detect large-scale chromosomal abnormalities rather than to perform high-resolution genotyping like in diagnostic settings. Commonly used CNV analysis tools remain robust at this level, and accepting a 95% threshold ensures a balance between data quality and resource efficiency in routine stem cell research.

The B-allele frequency (BAF) represents the genotype and is determined by the ratio of signal intensity from the B allele, which indicates the relative quantity of the one allele compared to the other. Homozygous SNPs exhibit BAFs close to 0 (AA) or 1 (BB), whereas heterozygous two-copy SNPs have BAFs near 0.5 (AB). Allelic imbalance leads to intermediate values. For instance, in a cell line with triploidy of a certain chromosome, an SNP is represented by three copies, which can have four possible genotypes (AAA, AAB, ABB, or BBB), resulting in BAFs of 0, 0.33, 0.66, or 1, respectively. In the case of a deletion, BAF shows values similar to those of the A/A and B/B genotypes, but no value for the A/B genotype (Attiyeh et al., 2009).

BAF drift quantifies deviations in the BAF from expected values (0, 0.5, or 1) in normal diploid regions. A drift of <0.01 is a benchmark for high-quality SNP data in sensitive analyses (Marenne et al., 2012; Rao et al., 2016; Taghizadeh et al., 2022). It can signal technical issues like poor DNA quality, sample degradation, or hybridization errors, leading to inaccurate genotyping. However, chromosomal aberrations can shift expected BAF positions, and GenomeStudio adjusts for these changes. In such cases, high BAF drift may indicate genomic instability, as observed in cancer cell lines, rather than technical flaws. Notably, GenomeStudio does not display BAF drift, making direct evaluation impossible.

The total fluorescence intensity is used to calculate the log R ratio (LRR). Each SNP probe on the array produces a signal intensity for the DNA sample, representing the observed intensity (R_observed_) of both alleles (R_A_ + R_B_). Additionally, a reference intensity R_expected_ is determined for each SNP, derived from a normalized dataset of many samples with a known, normal diploid genome. The LRR is calculated as follows:

$$LRR=\log_{2}\;\left(\frac{R_{observed}}{R_{expected}}\right).$$

The logarithmic transformation in LRR is used to normalize the data, making it easier to interpret copy number changes across a wide range of values. In a log_2_ scale, each unit corresponds to a doubling or halving of the copy number. The LRR with a value of 0 represents the presence of two chromosomes [log_2_ (1) = 0], representing the normal state. Elevated signal intensity of a region results in an increase in LRR, which represents copy number gain due to amplification or duplication [log_2_ (2) = 1, representing doubling]. A decrease in signal intensity is regarded as deletion, resulting in an LRR of approximately −1 [log_2_ (0.5) = −1, representing halving] for a hemizygous deletion with only one copy of a region, rather than the normal two copies (Peiffer et al., 2006).

The log R ratio standard deviation (LRR SD) is a key quality control metric in SNP array data analysis, automatically calculated using GenomeStudio. It estimates the overall noise in the data, where a lower LRR SD indicates higher data quality and reliability in detecting chromosomal aberrations. The typical threshold for LRR SD is 0.35. Samples below this value are considered to have good-quality data and are suitable for further analysis. For instance, an LRR SD below 0.2 is often desirable for precise and robust CNV calls. Samples with an LRR SD greater than 0.35 should be excluded from further analysis. Potential reasons for an elevated LRR SD include sample quality issues such as DNA degradation, contamination, or low DNA concentration; experimental factors like hybridization problems, batch effects, or array defects; and biological causes such as mosaicism or high variability in copy number across the genome, as observed in certain cancer cell lines (Bae et al., 2010; Marenne et al., 2012; Staaf et al., 2008).

The size of detected CNVs is an important value to ensure the quality of reported aberrations. Various studies have demonstrated the relationship between CNV size and detection accuracy for identifying genetic alterations. For example, the risk of false positives decreases as the CNV size increases. It is more difficult to distinguish smaller regions from noise or normal variations, whereby the analysis of larger regions is more significant and reliable (Canham et al., 2015). SNP array-based CNV calling algorithms, such as those used in cnvPartition, often miss CNVs smaller than 300 kb. The frequently cited threshold of 350 kb is based on a balance between sensitivity (the ability to detect true CNVs) and specificity (the ability to exclude false positives) (Lavrichenko et al., 2021; Soster et al., 2023; Zhang et al., 2014). CN-LOH is reported for regions of 1 Mb or larger because smaller regions have a higher risk of being false positive.

The CNV confidence value refers to a statistical measure that indicates the reliability or certainty of the detected CNV event and is crucial for distinguishing true CNVs from false positives. For instance, a confidence value above 70 for a called CNV is often considered to indicate a high probability that the CNV is real; scores below 70 may suggest a higher chance of the CNV being a false positive (Lin et al., 2011). We use a CNV confidence threshold of 70 or higher for our analysis. However, according to the literature, a threshold of 40 or higher is also commonly used, depending on the desired stringency of the analysis.

In the detection of CNVs, the minimum probe count defines the lowest number of consecutive array probes that must support a change in signal intensity for a CNV to be reported. This threshold helps distinguish true genomic alterations from background noise or technical variation. By requiring a minimum number of probes to indicate, for example, a duplication or deletion, the analysis software increases the reliability of CNV detection, particularly in regions of the genome where probe density or signal quality may vary. The cnvPartition plug-in uses a default minimum probe count of 3, but this threshold is adjustable and can be increased. In the literature, the minimum probe count for CNV detection is often set between 3 and 7 probes (Mason-Suares et al., 2013; Metzger et al., 2013; Urnikyte et al., 2016; Zhou et al., 2022). However, in hPSC research or in a more stringent context such as diagnostic application, thresholds of 10 or more probes are commonly used, as the risk of false-positive CNV calls increases significantly with lower probe counts (Laurent et al., 2011; Lin et al., 2013; Rajagopalan et al., 2020). In line with this, we applied a minimum probe count of 10 as part of the quality control process for our hPSC lines.

### Step-by-step workflow for quality control of hPSCs via GenomeStudio

**FIGURE 1:**
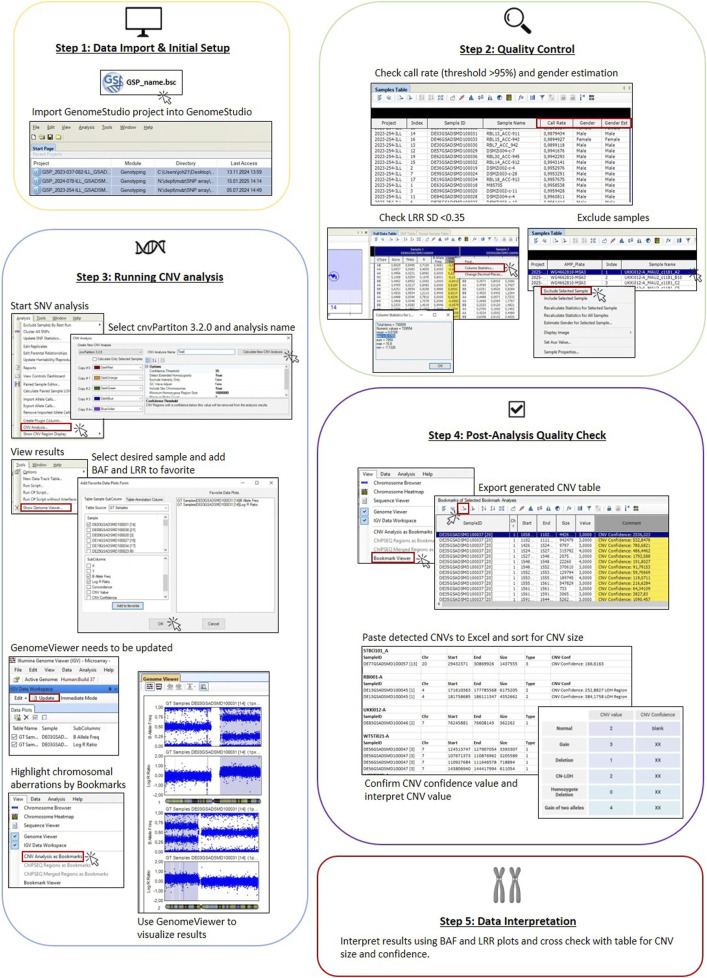
Workflow of SNP array analysis in GenomeStudio. Five steps in the workflow are indicated in boxes. Step 1 includes software setup and data import. Step 2 shows checks to be done for quality control of the data (call rate, gender estimate, and LRR SD) and how to exclude samples not passing QC. Step 3 shows how to run the analysis, covering selection of samples and BAF and LRR plots and displaying results in the Genome Viewer, including setting of bookmarks. Step 4 highlights the export of result table and post-analysis QC (size of CNV and CNV confidence value). Step 5 indicates reporting of results by cross-checking BAF and LRR plots.

Figure 1 represents all steps necessary for SNP array analysis using GenomeStudio to detect chromosomal aberrations.

#### Step 1 (a): data import and initial setup

To analyze SNP array data produced on a Global Screening Array v3.0 (GSAMD24v3-0, Illumina, Inc. San Diego) BeadChip, we used GenomeStudio v2.0.5, which is a free software without a licensing mechanism. GenomeStudio Installer can be downloaded from the Illumina support page together with the cnvPartition plug-in (https://emea.support.illumina.com/array/array_software/genomestudio/downloads.html). After downloading the software, only a one-time login to the Illumina account is necessary.

If the GenomeStudio project has already been created by the array facility, it can be easily loaded into GenomeStudio software by either using the ‘File’ menu and selecting ‘Open Project’ or simply dragging and dropping the GenomeStudio project (‘.bsc’) file into the GenomeStudio *start page* and proceeding with step 2.

#### Step 1 (b): creating a GenomeStudio project

If the GenomeStudio project needs to be created, the following files are essential: raw data files of each sample (.idat), manifest_file.bpm, cluster_position_file.egt, and the samplesheet.csv. The .idat files are raw data files encoding the measured fluorescence intensity of each probe. The manifest file contains information about the SNPs, their position, and probe sequences for the genotyping array, and it is mandatory for the analysis. Using a standard Illumina array, the official .bpm file is provided by Illumina or can be obtained by contacting Illumina technical support. We used the GSAMD-24v3-0-EA_20034606_A1.bpm manifest file. It should be noted that the BeadChips GSAMD24v3-0 and GSA-24v3.0 are not the same. Both refer to version 3 of the Global Screening Array (GSA) of Illumina. However, array type GSAMD24v3-0 contains approximately 50,000 additional SNPs that are related to multiple diseases (MDs). Care must be taken to select the correct manifest files as they are different for the two array types. In addition, the cluster position file (.egt) is provided by Illumina. The cluster position file is critical for accurate genotype calling; using the wrong .egt file can cause incorrect genotype calls and high no-call rates. It is important to use the .bmp and .egt files that match the used BeadChip. The samplesheet.csv is generally provided by the array facility and links the raw data (.idat) to the sample metadata, providing the sample ID, their location on the chip, and the used genotyping array. Using the sample sheet to create a new GenomeStudio project enables automatic assignment of sample IDs to .idat files for import. It is important to verify that the SentrixBarcodes_A are formatted in numbers (no decimal places) and that the manifest file name fits with the name on the sample sheet.

After saving the needed files, the GenomeStudio project can be created by starting GenomeStudio 2.0, using the *new project* button and electing *Genotyping* (standard setting for diploid organisms; Polyploid Genotyping is designed for organisms with more than two copies of each chromosome, like some plant or fish species). The Genotyping Project Wizard window opens and *project repository* and *project name* can be assigned. By clicking next, *use sample sheet to load sample intensities* can be selected, and in the next step, the directory of the *samplesheet*, the *raw data files* (including the .idat files), and the *manifest file* repository can be selected. The next step includes the import of the cluster positions from a cluster file giving the directory of the saved .egt file. After pressing finish, the data are getting normalized automatically, and the GenomeStudio project will be created.

#### Step 1 (c): sample size

To improve reliability, the sample size per GenomeStudio project should be at least five samples. However, this can be adjusted over time as more samples are analyzed. After collecting different GenomeStudio projects, it is recommended, but not mandatory, to eventually combine all samples into one large GenomeStudio project. This approach can help to detect recurrent or significant chromosomal aberrations, increasing confidence in CNV calls and reducing the likelihood that a detected aberration is a random artifact (Winchester and Ragoussis, 2012; Zhang et al., 2014). When creating a large GenomeStudio project, all data in the raw data folders from all samples should be integrated into a single folder, and a single sample sheet needs to be created. Then, a new GenomeStudio project should be generated, as explained in step 1 (b).

#### Step 2: quality control and sample exclusion

Call rate: after opening the GenomeStudio project, the call rate is included into the ‘sample table’ in addition to the project name, sample ID, and the sample name. If the call rate is too low (below 95), the sample should be excluded from further analysis.

Gender estimation: the expected and estimated gender is also included into the sample table for each sample, allowing the verification that the gender has been assigned correctly to each sample. If there is a mismatch between the expected and estimated gender, it could indicate a sample mix-up. However, certain chromosomal aberrations, such as the loss of the Y chromosome in male cell lines, can also lead to misclassification of gender. In such cases, the BAF and LRR plots need to be analyzed to determine the cause of the incorrect gender estimation.

LRR SD is an additional quality metrics, automatically calculated using GenomeStudio for each sample. The metric can be generated for each sample in the full data table by right-clicking on the column Log R ratio. Then, the column statistics is selected, and after calculation, the column statistics window opens. The LRR SD is displayed as *dev* value. Values for LRR SD under 0.35 are generally acceptable, whereas higher values may indicate increased noise in the data.

If any of the quality metrics fall outside the acceptable range, the sample should be excluded from further analysis. In such a case, isolation of genomic DNA and the SNP array need to be repeated. To exclude samples from CNV analysis, the individual sample should be selected in the “sample table,” and after right-clicking, *Exclude selected sample* can be selected. GenomeStudio asks whether the SNP statistics should be updated for all SNPs based on the remaining samples (click Yes).

#### Step 3: running CNV analysis

To perform CNV analysis, the cnvPartition 3.2.0 plug-in needs to be installed. By selecting *Analysis*–*CNV Analysis*, the *Create New CNV Analysis* window will open. cnvPartition 3.2.0 can be selected in this window, and a CNV Analysis Name can be specified. In the *Options table*, the minimum probe count can be set to 10. The analysis starts by pressing *Calculate New CNV Analysis*. Depending on the sample size, the process could take some time.

Once completed, the results can be viewed under *Tools*–*Genome Viewer*. The Illumina Genome Viewer will open alongside the *Add Favorite Data Plots Form*, where the desired sample and also BAF and LRR plots can be selected by *Add to favorite*. After closing the *Add Favorite Data Plots Form*, the Genome Viewer needs to be updated by using the *Update* button in the *IGV Data Workspace*. The *Genome Viewer* window provides two different modes to present the chromosomes: *Chromosome Slide Show Mode*, where each individual chromosome is depicted, choosing the desired chromosome by the arrow keys, or the *Whole Genome View Mode*, where all chromosomes are depicted in one large overview. The mode can be changed using the icons in the upper left corner of the *Genome Viewer* window. To inspect specific chromosomes and corresponding genes in detail, the Chromosome Browser can be selected by *View–Chromosome Browser* in the *Illumina Genome Viewer (IGV)–Microarray-*window.

For easier detection of CNVs, the *Bookmark viewer* can be accessed through *View*–*CNV Analysis as Bookmarks* in the *Illumina Genome Viewer (IGV)–Microarray-*window to highlight chromosomal aberrations by different colors to get a better overview (yellow = hemizygous deletion, green = copy-neutral LOH, and blue = duplication).

#### Step 4: post-analysis quality control

After running CNV analysis, it is important to sort the generated CNV table by the size of each called CNV to filter for the CNV size threshold. The list of all detected CNVs, along with chromosomal position, size, CNV confidence value, and copy number value (CNV value), is listed in a table under *View–Bookmark Viewer*. The Bookmark Analyses window will open. In the upper half of the window, the desired CNV analysis file is selected, and the table with the detected CNVs will open in the lower half of the window. To export the CNV analysis table as .txt file, click the export icon above the table (third from the left), and choose the location to save it. Open an Excel worksheet, and drag the .txt file into it. All CNVs with a size smaller than 350 kb should be removed from the table. After evaluating the CNV size, the remaining CNVs should be controlled for CNV confidence value. A CNV confidence value above 70 indicates a high probability that the CNV is real; scores below 70 may suggest a higher chance of the CNV being a false positive, and such CNVs need to be excluded.

The CNV table also includes the copy number value (CNV Value), which should not be confused with the CNV confidence value. The CNV Value indicates the quantity of chromosomal copies at a specific position. A CNV Value of 2 with a blank CNV confidence value signifies a normal diploid state. However, a CNV Value of 2 combined with a CNV confidence value above 70 suggests copy-neutral LOH. A CNV Value of 3 indicates an amplification, whereas a value of 1 represents a deletion. A CNV Value of 0 indicates a homozygous deletion, whereas a CNV Value of 4 represents a gain of two alleles. To validate these findings, it is essential to review the BAF and LRR plots, as described in step 3.

#### Step 5 (a): reporting chromosomal aberrations in hPSCs

After fulfilling all quality control criteria, detected CNVs can be reported as chromosomal aberrations. When analyzing SNP array data from reprogrammed, newly generated, or CRISPR/Cas9-edited hPSC lines, it is essential to compare the findings to those of the parental cell line. This comparison distinguishes newly acquired chromosomal abnormalities from pre-existing aberrations, identifying changes that may have occurred during reprogramming, gene editing, or cultivation. Clones with new chromosomal abnormalities can thus be excluded from further experiments, ensuring the genomic integrity of selected lines. Typically, multiple clones from a single parental line are generated post-reprogramming or after gene editing, which facilitates the selection of optimal clones. Clones with inconclusive SNP array results can be readily excluded, simplifying the identification of the most stable lines. It is also important to analyze and compare early DNA samples from hPSC lines to DNA harvested at later stages while banking hPSCs. This allows for the identification of any newly emerged chromosomal aberrations over time, preventing the use of clones with potential genomic instability.

When identifying chromosomal aberrations via the SNP array, it is crucial to carefully assess all quality control metrics and thresholds while thoroughly examining the BAF and LRR plots. Cross-referencing these metrics provides a comprehensive view of sample quality and helps distinguish true chromosomal aberrations from random artifacts, ensuring the reliability of the reported findings.

#### Step 5 (b): cross-referencing CNVs with the Database of Genomic Variants in the absence of parental material

In cases where no parental cell line material is available for comparison, the detected copy number variants (CNVs) can be cross-referenced with the Database of Genomic Variants (DGV). The DGV provides a publicly accessible and curated catalog of structural variation (SV) found in the genomes of control individuals from global populations. The database includes information on the location, size, and frequency of CNVs (MacDonald et al., 2014; Park et al., 2011; Shaikh et al., 2009). By comparing the detected CNVs to the DGV, it is possible to distinguish common, benign CNVs that are likely already present in the parental cell line from those that may be newly introduced during gene editing or banking.

The DGV website is accessible via http://dgv.tcag.ca/dgv/app/home and provides a search function to enter the specific CNV of interest by genomic coordinates. The search result will display a list of CNVs matching the chromosomal region with detailed information about the CNV. The included frequency will indicate how common the CNV is in the normal population, often presented as a percentage.

#### Step 5 (c): segmental duplications

Segmental duplications (SDs) are large blocks of DNA (typically >1 kb in size) that occur more than once in the human genome. The repetitive sequence content in these areas can lead to cross-hybridization artifacts and unreliable signal intensities, increasing the likelihood of false-positive CNV calls. To reduce misinterpretation, detected duplications should be cross-referenced with a publicly available SD annotation track, such as the one provided by the UCSC Genome Browser (Abdullaev et al., 2021; Bailey et al., 2001; Bailey et al., 2002; Sharp et al., 2005). CNVs overlapping these regions should be flagged by the user and may be excluded from reporting unless they are related with well-known recurrent abnormalities in hPSCs. For example, gains at chromosome 20q11.21 are often detected as culture-acquired alterations in hPSCs, which partially overlap SDs. However, these should not be dismissed as they represent biologically significant changes (Halliwell et al., 2021; Jeong et al., 2025).

#### Troubleshooting

In practice, deviations in BAF and LRR plots can be early indicators of technical problems. If the plots appear noisy or inconsistent, like a high LRR SD (showing a very broad signal in the LRR plot) and the BAF plots show substantial scatter, it may indicate issues with normalization efficiency. In such cases, we recommend recreating the GenomeStudio project from the raw .idat files. This triggers a new normalization process, which can sometimes resolve the issue if the problem stems from project corruption or incorrect initial settings (Supplementary Figures S1A,B). If re-normalization does not improve the data, a technical failure of the array or compromised sample quality should be considered. At that point, repeating the SNP array, ideally with freshly quantified and quality-controlled genomic DNA, is advisable.

If all BAF plots in the Genome Viewer display additional allele frequency signal clusters beyond the expected 0, 0.5, and 1, this indicates a mix of two or more samples, resulting in more than two alleles at many SNP positions. The number of signals increases with the number of contaminating samples. Although the analysis can detect contamination, it cannot identify its source (Supplementary Figure S1C).

## Results

### Data interpretation using BAF and LRR plots

**FIGURE 2:**
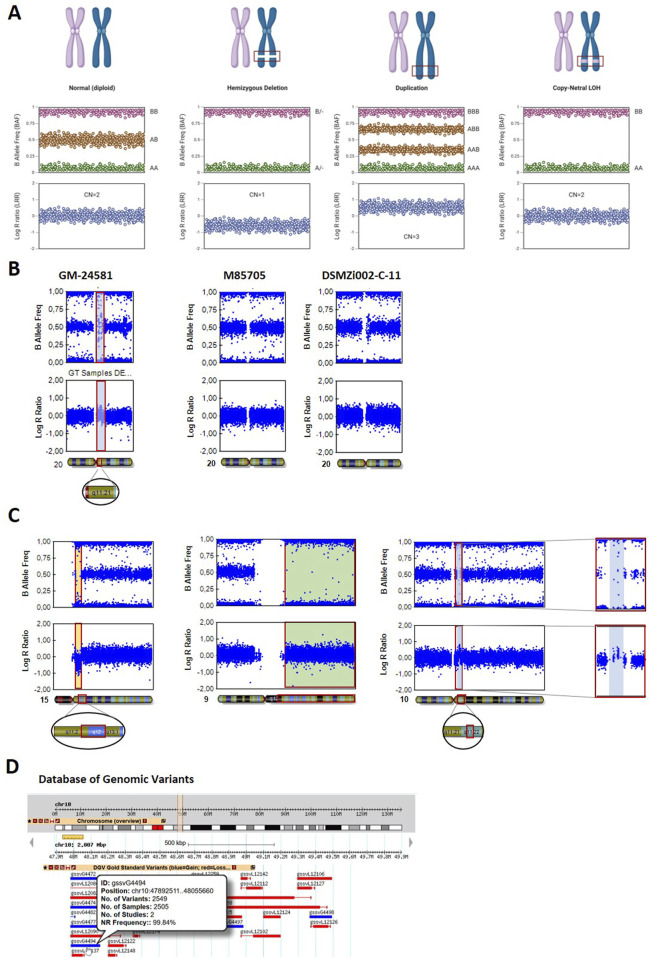
Detection of chromosomal aberrations using BAF and LRR plots. **(A)** Appearance of BAF and LRR plots for different chromosomal states, including the normal diploid state, hemizygous deletion, duplication, and CN-LOH. Created in BioRender https://BioRender.com/idh2c56. **(B)** Common duplication of chr20q11.21 in the hiPS cell line GM24581, highlighted in blue using a bookmark. **(C)** Chromosomal aberrations detected via GenomeStudio in cell line DSMZi017-A. A deletion in the chromosome 15q11–q13 region is depicted in yellow (left panel). A CN-LOH affecting the entire long arm of chromosome 9 is highlighted in green (middle panel). A small gain on chromosome 10q11.22 is marked in blue (right panel). **(D)** The detected chromosome 10q11.22 duplication is compared with data from the Database of Genomic Variants (DGV). The CNV gssvG4494, which fully spans the duplication, is highlighted.

The BAF and LRR plots are essential for detecting chromosomal aberrations via the SNP array, showing specific patterns based on the genotype (BAF) and signal intensity (LRR) (Figure 2A). A normal, diploid set of chromosomes shows clear BAF clustering at 0 (AA), 0.5 (AB), and 1 (BB), representing a typical heterozygous SNP pattern. The LRR plot shows a horizontal line of signal clusters around 0, indicating two copies of the chromosome.

For a hemizygous deletion (one copy loss), the BAF plot lacks the heterozygous signal cluster at 0.5 (no heterozygous SNPs) and shows only two signal clusters around 0 and 1, representing homozygous AA or BB genotypes. The LRR plot shows a negative shift in the LRR (around −0.5 to −1), indicating a reduction in the copy number.

In duplications (one extra copy), the BAF plot displays signal clusters between 0 and 0.5 (at 0.33) and between 0.5 and 1 (at 0.66), reflecting the presence of three possible alleles in four genotypes (AAA, AAB, ABB, and BBB), and the LRR plot shows a positive shift (around +0.3 to +0.5), indicating an increase in the copy number.

Finally, CN-LOH, either due to uniparental disomy or chromosomal loss followed by duplication of the remaining copy, shows a complete loss of heterozygosity in the BAF plot, showing only two signal clusters: 0.0 (AA) and 1.0 (BB). There is no middle cluster at 0.5 (AB), which would indicate heterozygosity. The LRR plot remains close to zero because there is no change in the overall copy number. The region is still diploid (two copies of the chromosome), so no gain or loss of genetic material is reflected in LRR.

It is important to use both, BAF and LRR plots, when analyzing chromosomal aberrations because events like a deletion and a CN-LOH can appear identical in the BAF plot but differ in the LRR plot.

### Examples of in-house detected chromosomal variants in hPS cell lines

We analyzed a total of 40 different hPSC lines for chromosomal integrity using the SNP array (Supplementary Table S1) and detected chromosomal aberrations in nine independent lines (10.9%) (Table 1).

The gain of 20q11.21 is one of the most common aberrations in iPSCs and was detected in three independent iPS cell lines: ZIPi015-K, GM24581, and STBCi101-A. The aberration most likely appeared during cell cultivation (Amps et al., 2011). Amplification of 20q11.21 is visible in the BAF plot as data points clustering around 0.33 and 0.66, along with an increase in signal intensity at this position in the LRR plot, shown for iPSC line GM24581 (Figure 2B, left panel). The same aberration was detected in one clonal line after reprogramming (DSMZi002-C-11), whereas the parental fibroblast culture (M85705) did not show this amplification (Figure 2B, right panel).

Another iPSC line (DSMZi017-A), used in a study for DNA methylation analysis, was derived from a patient with Angelman syndrome, characterized by a large deletion on the maternal chromosome 15q11–q13 (Chamberlain et al., 2010). This deletion was confirmed through SNP array analysis, which revealed a hemizygous deletion in the 15q11–q13 region (Figure 2C, left panel). The BAF plot displayed an absence of signals at the 0.5 value, whereas the LRR plot showed a clear reduction in the signal intensity in the same region. In addition, the SNP array identified a large region of copy-neutral loss of heterozygosity (CN-LOH) on the long arm of chromosome 9 (approximately 75 Mbp). This LOH was evident through a loss of signal in the BAF plot, whereas the LRR plot remained unchanged in this area (Figure 2C, middle panel). The detected CN-LOH probably appeared during cell culture due to the high passage number of the cell line. Additionally, a small gain was detected on chromosome 10q11.22, indicated by clusters of data points at 0.33 and 0.66 in the BAF plot and an increase in signal intensity in the LRR plot (Figure 2C, right panel). As the cell line was reprogrammed several years ago (Chamberlain et al., 2010), no parental material is available for comparison of the detected chromosomal aberration. Numerous gains of 10q11.22 have been reported in the DGV. The CNV gssvG4494, which spans the detected region (chr10:47892511…48055660), is reported with a population frequency of 99.84% (Figure 2D). This strongly suggests that the observed aberration was already present in the parental cell line as a normal genomic variant.

SNP array analysis of the human embryonic stem cell line H9 revealed a gain of 7q11.21, along with a gain in chromosomal region 14q23.3 (Table 1) and a loss of chromosomal region 16q11.2. Although gains of entire chromosomes 7 and 14 are commonly reported in hPSC lines, the specific regions described here have not been explicitly noted in these reports (Baker et al., 2016; Baker et al., 2007). No accordance of the specific positions of the observed gains and loss were found by comparison with the DGV, which could indicate that the aberrations occur during cell cultivation.

The iPSC line RBi001-A exhibited a CN-LOH at chromosome region 4q33–q34.3 (Table 1). Due to the lack of reference material, it remains unclear whether the detected aberration originated during cell culture or was already present in the parental cell line. Notably, CN-LOH at this locus has not been frequently reported in the literature as a common occurrence during hPSC culturing.

In the iPSC line WTSIi021-A, deletions were detected in regions 7q31.1 and 7q31.33 (Table 1). Because deletions affecting parts of the q-arm of chromosome 7 have been frequently described in the literature, we assume the deletion to be an adaptation to cell culture conditions (Andrews et al., 2022).

Cell line ZIPi015-K underwent two rounds of CRISPR/Cas9 gene editing, targeting chromosome 9 (ZIPi015-K-1) and subsequently chromosome 15 (Haake et al., 2024). A clonal cell line of the chromosome 9 editing, with no additional chromosomal aberrations than the 20q11.21 gain, which was already present in the parental cell line, was selected for gene editing, targeting chromosome 15. Among eight clonal cell lines screened post-editing, one exhibited a CN-LOH of the whole q-arm of the targeted chromosome 15 (ZIPi015-K-2-C-3) (Table 1).

### Detection of Xp isochromosome via G-banding and SNP array

**FIGURE 3:**
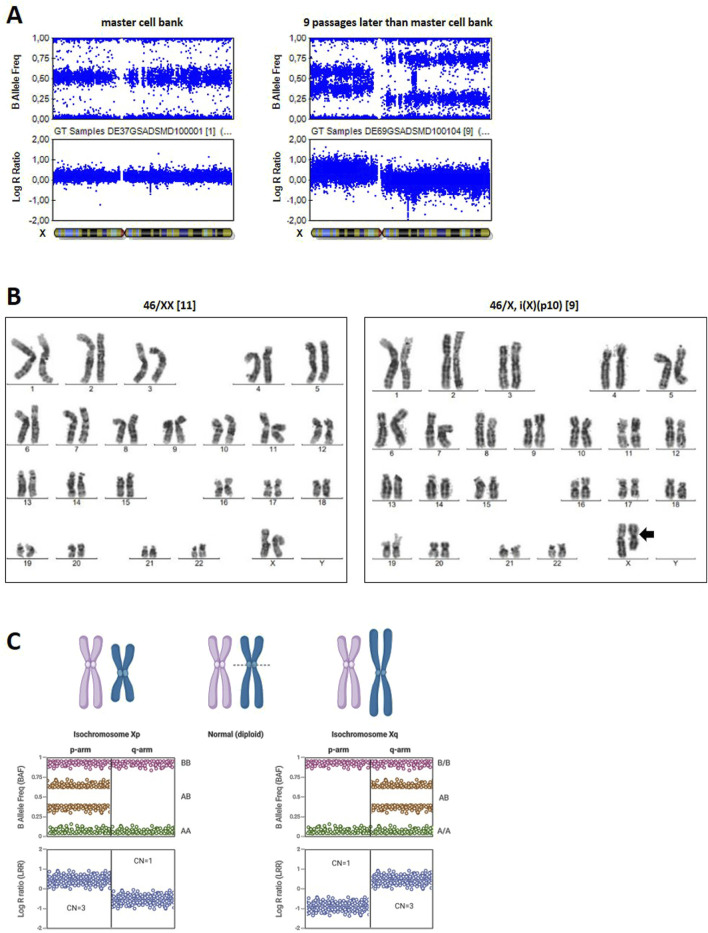
Karyogram of the Xp isochromosome. **(A)** BAF and LRR plots of the X-chromosome of cell line DSMZi017-A-2-A8 in SNP array analysis at the stage of master cell bank generation (left) and nine passages later during expansion for G-banding analysis (right). The Xp isochromosome is evident in the right plot. Mosaic state is visible because of additional signal clusters at the Xq arm. **(B)** the G-banding results of the cell line DSMZi017-A-2-A8, revealing the existence of two distinct cell populations: one with a normal chromosome formula of 46/XX (left panel) and one carrying an Xp isochromosome 46,/X, i(X) (p10) (right panel, isochromosome is indicated by an arrow), with the breakpoint near the centromere (p10). A total of 20 metaphases were analyzed, whereby 11 metaphases showed the normal chromosomal formula and in 9 metaphases, the isochromosome Xp was detected. **(C)** Schematic representation of BAF and LRR plots for Xp and Xq isochromosomes. Created in BioRender https://BioRender.com/ogtq4dp.

Cell line DSMZi017-A was also used for two rounds of CRISPR/Cas9 gene editing at chromosomes 9 (DSMZi017-A-1) and 15, followed by quality control including SNP array analysis and G-banding. Four clonal lines (DSMZi017-A-2-A3/-A8/-C12/-F4) were generated and analyzed via G-banding and SNP array within the quality control of establishing a master cell bank. Although the SNP array data did not show any abnormalities (Figure 3A left panel), G-banding analysis of clone DSMZi017-A-2-A8 exhibited two distinct cell populations: one with a normal karyotype, detected in 11 out of 20 metaphases, and another with an Xp isochromosome, detected in 9 out of 20 analyzed metaphases (Figure 3B). Isochromosomes are classified as structural chromosomal aberrations that are caused by improper segregation during cell division. This missegregation results in an unbalanced chromosomal complement, with the duplication of genetic material from one arm and the loss of material from the other. This results in a chromosome with two identical arms; in the case of an Xp isochromosome, both arms are short arms (p arms) (Chadwick, 2020; McFeely, 1993; Mertens et al., 1994). As cells for G-banding analysis were harvested 13 passages later than for the DNA of the master cell bank samples, we assumed that acquisition of the Xp isochromosome occurred during this extended time of culture. Indeed, it was detected via SNP array in a sample of genomic DNA prepared nine passages after the preparation of the master cell bank (Figure 3A, right panel). In the SNP array, an Xp isochromosome appears as duplication in the BAF plot (signal clusters at 0.33 and 0.66) and a positive shift in the LRR plot, reflecting extra p-arm copies (Figure 3C). In contrast, the region of the plot corresponding to the Xq arm would resemble a deletion in the BAF plot, characterized by the absence of signals at 0.5, and an increase in the LRR plot. For an Xq isochromosome, these patterns are reversed (Figure 3C). In the sample analyzed, the BAF plot of the Xp arm showed the expected pattern for duplication, with clusters around 0.33 and 0.66. However, for the Xq arm, instead of the clean deletion profile observed in a non-mosaic cell population carrying the Xp isochromosome, additional signal intensities around 0.25 and 0.75 were observed. This suggests a mixed cell population, as detected via G-banding, where the presence of both, normal cells and cells with the Xp isochromosome, leads to intermediate BAF values due to partial allelic imbalance (Eberhardt et al., 2023; Glessner et al., 2021; Conlin et al., 2010).

## Discussion

The presented findings highlight the frequency of chromosomal instability that can arise during reprogramming, gene editing, or extended cell culturing and banking. SNP array analysis complements traditional methods like G-banding, providing a higher resolution for detecting numerical variants. SNP array analysis provides a fast and cost-effective method to routinely verify genomic integrity of hPSC lines and can be incorporated as a critical component of quality control pipelines, applied throughout cell banking and clone selection processes. To enable SNP array analysis by researchers without profound expertise in bioinformatics, this practical guide was compiled. In setting up the analysis in our laboratory, we encountered several difficulties in finding and collecting all information needed to generate a GenomeStudio Project and to perform the analysis. To the best of our knowledge, there is no guide explaining the necessary steps to comprehensive analysis of SNP array data generated through Illumina BeadArray technology. However, only straightforward access to techniques and analysis will promote molecular karyotyping in the hPSC field, resulting in better QC, better data, and higher reproducibility.

Of course, alternative tools other than cnvPartition exist for SNP array analysis. One example is PennCNV, a free software application capable of handling Illumina signal intensity data with high sensitivity. PennCNV can detect CNVs down to ∼100 kb, depending on array resolution and data quality (Tsuang et al., 2010; Wang et al., 2007; Zhou et al., 2022). The increased sensitivity is due to the use of a different mathematical model, a Hidden Markov Model, which integrates LRR, BAF, and population frequency of alleles. In contrast, cnvPartition uses a likelihood-based recursive partitioning algorithm that primarily relies on LRR values, favoring larger CNVs while reducing the likelihood of false positives (Pinto et al., 2011; Winchester et al., 2009). Despite the higher sensitivity of PennCNV, we decided to use cnvPartition within GenomeStudio due to its straightforward integration with Illumina data and its robustness for detecting larger, potentially more relevant CNVs in the context of hPSC QC. Given its accessibility and user-friendly interface, cnvPartition remains a practical and effective choice for routine CNV assessment.

The findings of nine aberrations among 40 hPS cell lines analyzed in this study highlight the necessity of such analyses at a frequent interval during experimentation. The common duplication of chromosome 20q11.21 was observed in 4 out of 31 hPSC lines (12.9%; sub-clonal lines of ZIPi015-K were excluded in this calculation). In contrast, the International Stem Cell Initiative reported the same duplication in over 20% of analyzed iPSC lines (Amps et al., 2011). The discrepancy is likely attributable to the smaller number of cell lines analyzed in our study compared to the larger sample size in the International Stem Cell Initiative, which provides a more comprehensive representation of the frequency of this duplication. The minimal 20q11.21 gain encompasses the genes *ID1*, *BCL2L1*, and *HM13* and has frequently been observed to arise during PSC culture. The *BCL2L1* gene is linked to reduced susceptibility to apoptosis, which confers a growth advantage and promotes culture adaptation (Avery et al., 2013; Jo et al., 2020; Laurent et al., 2011; Markouli et al., 2019; Nguyen et al., 2014). Furthermore, *BCL2L1* overexpression alters TGF-β and SMAD-mediated signaling, negatively impacting neuroectodermal differentiation (Markouli et al., 2019). The impact of the alteration to culture adaptation and differentiation potential underscores the critical importance of chromosomal stability checks. Additionally, a gain involving a part of chromosome 14 has been noted, but in most cases, the literature describes gains of the entire chromosome 14 rather than partial gains in regard to cell culture adaption in hPSCs. The detected gain in the H9 cell line is located on chromosome 14q23.3, spanning a region that includes the *FUT8* gene. Fucosyltransferase 8 is an enzyme responsible for core fucosylation, a critical post-translational modification of N-glycans. Numerous studies have shown that FUT8 is abnormally overexpressed in various cancer types, leading to malignant transformations such as proliferation, invasion, and metastasis (Bastian et al., 2021; Shi et al., 2024). The described alteration could lead to selective growth advantage of the carrier cells, which may result in malignancy *in vivo* (Andrews et al., 2022). Deletions of parts of chromosome 7q were detected in cell line WTSIi021-A, which is also described in the literature to occur in hPSCs during cell culturing. One of the detected deletions on chromosome 7q31.33 includes, in addition to several other genes, *PAX4*. PAX4, a transcription factor from the PAX family, is essential for human endocrine and proper β-cell development and plays a crucial role in the differentiation of hPS cells into insulin-producing cells. Reduced PAX4 expression in hPS cells may affect their differentiation into insulin-producing β-cells negatively, which could be crucial for diabetes studies (Blyszczuk et al., 2003; Liew et al., 2008; Lin et al., 2007). Another gene located in the same region of the detected deletion (7q31.33) is *SND1*, which is involved in multiple biological processes, including RNA splicing, transcription, RNA-induced silencing complex (RISC), and RNA epigenetics. SND1 regulates endothelial function, and its inhibition leads to endothelial dysfunction (Han et al., 2025). For instance, the detected aberration could negatively impact studies using iPSC-derived endothelial cell models. Gains of chromosome 7q occur far more frequently than deletions, but the specific position of the detected gain of chromosome 7q11.21 in cell line H9 was not explicitly mentioned in previous reports (Andrews et al., 2022; Baker et al., 2016; Baker et al., 2007).

The extent of chromosomal abnormalities, especially when they span a larger area and affect multiple genes, is difficult to assess, highlighting the importance of excluding such cell lines from further experiments to avoid unintended impacts on experimental outcomes. Incorporating SNP array analyses into the routine quality control of hPSCs is therefore essential and should be conducted regularly. It is crucial to examine the early source material of a cell line to determine whether aberrations emerged during culture or were already present in the parental cell line. For example, comparing the original source cells with the reprogrammed iPSC line can provide valuable insights into the origins of aberrations. Similarly, after CRISPR/Cas9 gene editing, analyses should be performed to identify clonal lines without chromosomal abnormalities. Only such clones should be selected and established as cell lines for further use. We recommend to implement a cell banking system composed of master and working cell banks (MCB and WCB) that underwent standardized quality control according to the ISSCR standards (Ludwig et al., 2023). This system supports the use of standardized cell sources for a long time and contributes to reproducibility of results. CNV analysis at the time of generating the master cell bank is essential as this represents the earliest reference point for downstream applications. How often genomic integrity is examined during the generation and experimentation of WCB is at the discretion of the researcher. Re-testing the cells is recommended in cases of bottle-necking selection after genome editing or extended cultivation, specifically beyond 10 passages post-thaw, because genetic alterations are known to accumulate during prolonged *in vitro* expansion (Baker et al., 2007; Bai et al., 2013; Tigges et al., 2021). The emergence of an Xp isochromosome in one clonal line within nine passages after genome editing, as described here, highlights the importance of monitoring chromosomal integrity during long-term cultivation of hPSCs.

The combination of G-banding and molecular analysis is crucial for comprehensive quality control in hPSC generation and banking, as well as regular control of chromosomal integrity. Although SNP array remains a standard for detecting chromosomal abnormalities in hPSCs on the molecular level, low-pass whole-genome sequencing (lp-WGS) is emerging as a powerful alternative (Edwards et al., 2024; Jiang et al., 2025; Sarkar et al., 2021). Sequencing the genome at low coverage (0.1–1x) enables the detection of large-scale copy number alterations and aneuploidies (Chau et al., 2020), with the benefit of uniform genome-wide coverage not constrained by probe design. Analysis tools like QDNAseq and ACE, both implemented in R, support copy number alteration detection with visual outputs and user-friendly interfaces (Poell et al., 2019; Scheinin et al., 2014). Although lp-WGS requires sequencing infrastructure, it is becoming cost-competitive, making it a flexible option for routine genomic QC in hPSC workflows.

Regardless of the method used, testing genomic integrity is essential in QC of hPSCs, and the detailed workflow provided here reinforces SNP array as one reliable and practical tool for routine QC of hPSCs.

## Data Availability

Data of 15 published and/or publicly available hPSC lines analyzed in this study were deposited in the BioStudies database (https://www.ebi.ac.uk/biostudies/) under accession number S-BSST2033. The included raw data files (.idat files) are also accessible via ArrayExpress (https://www.ebi.ac.uk/biostudies/arrayexpress) under accession number E-MTAB-15176.
