# Supplementary material for: Chromosomal quality control in hPSCs: A practical guide to SNP array analysis with GenomeStudio
Source: Front Cell Dev Biol. 2025 Jul 1;13:1599923. doi: 10.3389/fcell.2025.1599923 (PMC12259632; doi:10.3389/fcell.2025.1599923)
Supplement: Supplementary file 1 [file Supplementaryfile1.pdf]

Supplementary Material

Table S1: Overview of in-house used cell lines and cultivation conditions. 40 iPSC lines were analyzed by SNP array. Abbreviations: hiPSC: human induced pluripotent stem cells, hESC: human embryonic stem cells, iPSBrew: StemMACS^{TM} iPS-Brew XF, human (Miltenyi Biotec, Bergisch Gladbach, Germany), mTeSR^{TM} Plus (Stemcell Technologies, Cologne, Germany), E8: Essential 8^{TM} Medium (ThermoFischer Scientific, Darmstadt, Germany), GCDR: Gentle Cell Dissociation Reagent (Stemcell Technologies, Cologne, Germany), EDTA was used at 0.5 mM (Thermo Fisher Scientific, Darmstadt, Germany). Matrigel was used at 0.017 mg/cm^2 (Corning by Merck, Darmstadt, Germany), Vitronectin^{TM} was used at 10 µg/ml (Stemcell Technologies, Cologne, Germany).

**Cell-line**	**Cell line type**	**Source**	**Medium**	**Surface coating**	**Passage method**
DSMZi001-A	hiPSC	reprogrammed	iPSBrew	Vitronectin	GCDR
DSMZi001-A-1	hiPSC	Haake et al., 2024	iPSBrew	Vitronectin	GCDR
DSMZi002-C-4	hiPSC	reprogrammed	iPSBrew	Vitronectin	GCDR
DSMZi002-C-11	hiPSC	reprogrammed	iPSBrew	Vitronectin	GCDR
DSMZi002-C-16	hiPSC	reprogrammed	iPSBrew	Vitronectin	GCDR
DSMZi003-C-10	hiPSC	reprogrammed	iPSBrew	Vitronectin	GCDR
DSMZi003-C-28	hiPSC	reprogrammed	iPSBrew	Vitronectin	GCDR
DSMZi003-C-38	hiPSC	reprogrammed	iPSBrew	Vitronectin	GCDR
DSMZi004-C-3	hiPSC	reprogrammed	iPSBrew	Vitronectin	GCDR
DSMZi004-C-4	hiPSC	reprogrammed	iPSBrew	Vitronectin	GCDR
DSMZi004-C-7	hiPSC	reprogrammed	iPSBrew	Vitronectin	GCDR
DSMZi017-A (ASdel1-0)	hiPSC	Chamberlain et al, 2010	iPSBrew	Vitronectin	GCDR
DSMZi017-A-1	hiPSC	Haake et al., 2024	iPSBrew	Vitronectin	GCDR
DSMZi017-A-2-A3	hiPSC	gene edited	iPSBrew	Vitronectin	GCDR
DSMZi017-A-2-A8	hiPSC	gene edited	iPSBrew	Vitronectin	GCDR
DSMZi017-A-2-C12	hiPSC	gene edited	iPSBrew	Vitronectin	GCDR
DSMZi017-A-2-F4	hiPSC	gene edited	iPSBrew	Vitronectin	GCDR
GM24581	hiPSC	Corriell repository	iPSBrew	Vitronectin	GCDR
H9	hESC	Kanber et al., 2022	mTeSR	Matrigel	EDTA
H9-C07	hESC	Kanber et al., 2022	iPSBrew	Matrigel	GCDR
H9-G12LS	hESC	Kanber et al., 2022	iPSBrew	Matrigel	GCDR
iPS(IMR90)-4	hiPSC	WiCell	mTeSR	Matrigel	EDTA
NCUi001 (RB1het)	hiPSC	Rozanska et al., 2019	mTeSR	Matrigel	EDTA
NCUi001-A (RB1wt)	hiPSC	Rozanska et al., 2019	mTeSR	Matrigel	EDTA
NCUi001-B (RB1ko)	hiPSC	Rozanska et al., 2019	mTeSR	Matrigel	EDTA
RBi001-A	hiPSC	EBiSC	iPSBrew	Vitronectin	EDTA
STBCi101-A	hiPSC	EBiSC	mTeSR	Matrigel	GCDR
UKKi012-A	hiPSC	EBiSC	E8	Vitronectin	EDTA
WTSIi021-A	hiPSC	EBiSC	E8	Vitronectin	EDTA
WTSIi075-A	hiPSC	EBiSC	E8	Vitronectin	EDTA

ZIPi015-K	hiPSC	Neureiter et al., 2015	iPSBrew	Vitronectin	GCDR
ZIPi015-K-1	hiPSC	Haake et al., 2024	iPSBrew	Vitronectin	GCDR
ZIPi015-K-2-C-1	hiPSC	gene edited	iPSBrew	Vitronectin	GCDR
ZIPi015-K-2-C-2	hiPSC	gene edited	iPSBrew	Vitronectin	GCDR
ZIPi015-K-2-C-3	hiPSC	gene edited	iPSBrew	Vitronectin	GCDR
ZIPi015-K-2-C-4	hiPSC	gene edited	iPSBrew	Vitronectin	GCDR
ZIPi015-K-2-C-5	hiPSC	gene edited	iPSBrew	Vitronectin	GCDR
ZIPi015-K-2-C-6	hiPSC	gene edited	iPSBrew	Vitronectin	GCDR
ZIPi015-K-2-C-7	hiPSC	gene edited	iPSBrew	Vitronectin	GCDR
ZIPi015-K-2-C-8	hiPSC	gene edited	iPSBrew	Vitronectin	GCDR

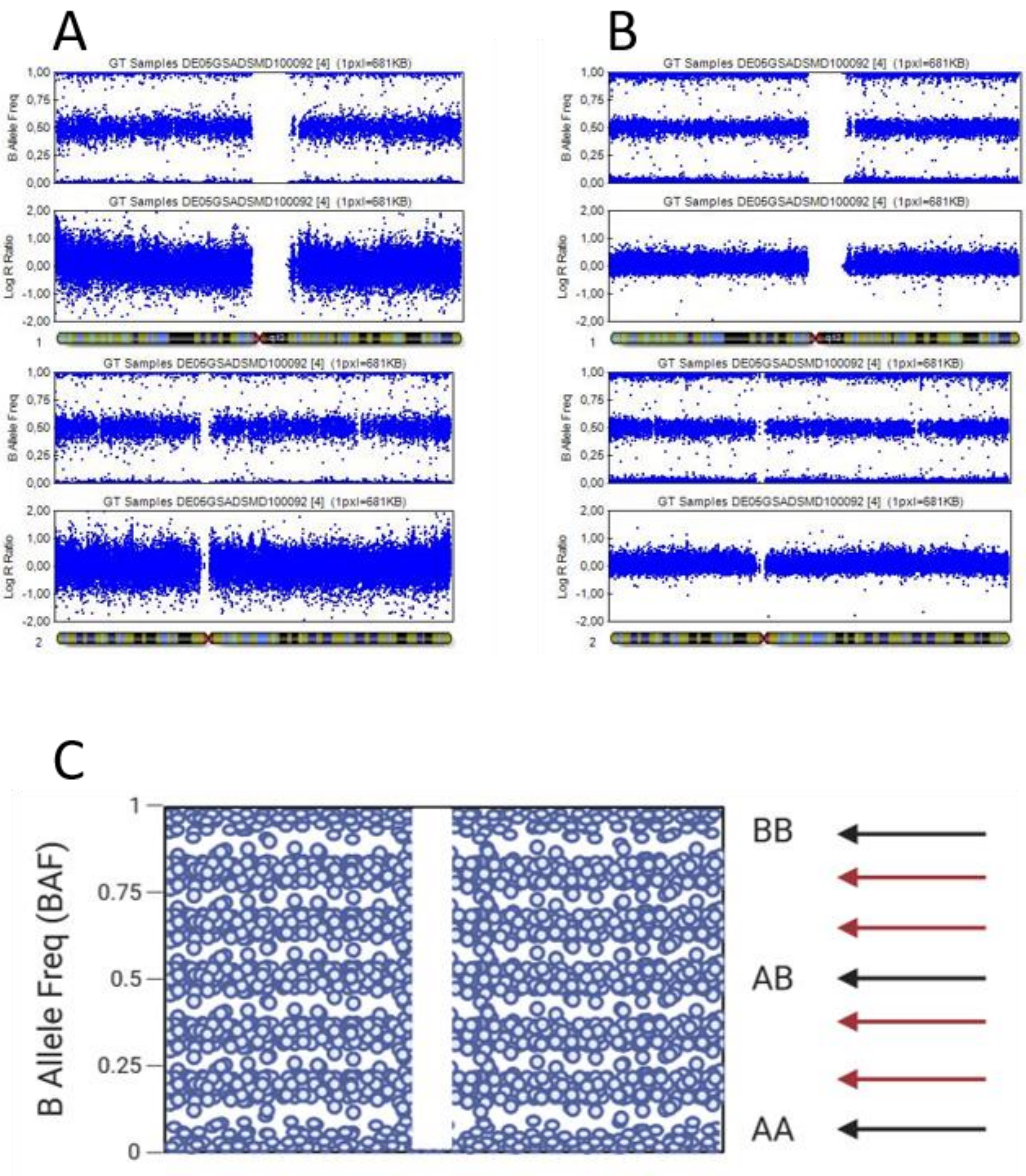

Figure S1. Improvement of data quality by re-normalization and detection of cross-contamination. A) Plots of chromosome 1 and 2 showing substantial scatter in the BAF plot and high variability in LogR Ratios. B) Plots of the same sample data than in A, after re-normalization by generation of a new GenomeStudio project. C) Schematic B allele frequency (BAF) plot illustrating cross-contamination from three samples. Black arrows indicated the expected signal clusters of a diploid sample (0: AA; 0.5: AB; 1: BB). Red arrows point to additional signal clusters due to the mixing of allelic signals from multiple genomes. The contamination signature is expected to appear across all chromosomes in the affected sample. Created in BioRender https://BioRender.com/dz8ppvg.

Supplementary references

Kanber, D., Woestefeld, J., Döpper, H., Bozet, M., Brenzel, A., Altmüller, J., Kilpert, F., Lohmann, D., Pommerenke, C., & Steenpass, L. (2022). RB1-Negative Retinal Organoids Display Proliferation of Cone Photoreceptors and Loss of Retinal Differentiation. *Cancers, 14(9), 2166*. https://doi.org/10.3390/cancers14092166

Neureiter, A., Brändl, B., Hiber, M., Tandon, R., Müller, F. J., & Steenpass, L. (2018). Generation of an iPSC line of a patient with Angelman syndrome due to an imprinting defect. *Stem cell research*, *33, 20–24*. https://doi.org/10.1016/j.scr.2018.09.015

Rozanska, A., Cerna-Chavez, R., Queen, R., Collin, J., Zerti, D., Dorgau, B., Beh, C. S., Davey, T., Coxhead, J., Hussain, R., Al-Aama, J., Steel, D. H., Benvenisty, N., Armstrong, L., Parulekar, M., & Lako, M. (2022). pRB-Depleted Pluripotent Stem Cell Retinal Organoids Recapitulate Cell State Transitions of Retinoblastoma Development and Suggest an Important Role for pRB in Retinal Cell Differentiation. *Stem cells translational medicine, 11(4),* 415–433. https://doi.org/10.1093/stcltm/szac008
